# Calcium oxalate crystals increased enolase-1 secretion from renal tubular cells that subsequently enhanced crystal and monocyte invasion through renal interstitium

**DOI:** 10.1038/srep24064

**Published:** 2016-04-05

**Authors:** Wararat Chiangjong, Visith Thongboonkerd

**Affiliations:** 1 Medical Proteomics Unit, Office for Research and Development, Faculty of Medicine Siriraj Hospital, Mahidol University, Bangkok, Thailand; 2 Graduate Program in Molecular Medicine, Faculty of Science, Mahidol University, Bangkok, Thailand; 3 Center for Research in Complex Systems Science, Mahidol University, Bangkok, Thailand

## Abstract

Calcium oxalate monohydrate (COM) crystals cause kidney stone disease by still unclear mechanisms. The present study aimed to characterize changes in secretion of proteins from basolateral compartment of renal tubular epithelial cells after exposure to COM crystals and then correlated them with the stone pathogenesis. Polarized MDCK cells were cultivated in serum-free medium with or without 100 μg/ml COM crystals for 20 h. Secreted proteins collected from the lower chamber (basolateral compartment) were then resolved in 2-D gels and visualized by Deep Purple stain (n = 5 gels/group). Spot matching and intensity analysis revealed six protein spots with significantly altered levels in COM-treated samples. These proteins were then identified by tandem mass spectrometry (Q-TOF MS/MS), including enolase-1, phosphoglycerate mutase-1, actinin, 14-3-3 protein epsilon, alpha-tubulin 2, and ubiquitin-activating enzyme E1. The increased enolase-1 level was confirmed by Western blot analysis. Functional analysis revealed that enolase-1 dramatically induced COM crystal invasion through ECM migrating chamber in a dose-dependent manner. Moreover, enolase-1 bound onto U937 monocytic cell surface markedly enhanced cell migration through the ECM migrating chamber. In summary, our data indicated that the increased secretory enolase-1 induced by COM crystals played an important role in crystal invasion and inflammatory process in renal interstitium.

Renal tubular epithelial cell is one of the polarized cells composed of apical and basolateral compartments, which can secrete a set of compartment-specific proteins that play important roles in homeostasis and pathophysiological processes. Changes in these secreted proteins during various diseases and stages, thus, can serve as biomarkers for diagnostics and prognostics and may lead to better understanding of the disease mechanisms^1^. Most of the secreted proteins from apical compartment of renal tubular epithelial cells are involved in membrane biology and ion transport^2^,^3^, whereas those from basolateral compartment are mainly associated with signaling cascade, inflammatory response, and hormone/reproductive system^4^,^5^.

In kidney stone disease, the most common pathogenic crystal found in stone matrices obtained from stone formers is calcium oxalate monohydrate (COM)^6^. Interaction between COM crystals and renal tubular epithelial cells causes changes in cellular proteins that can be linked to some pathogenic mechanisms of kidney stone formation^7^,^8^. We have hypothesized that alterations in secreted products from renal tubular epithelial cells after exposure to COM crystals also play significant roles in the stone pathogenesis. However, changes in these secretory products remained unknown. The present study thus aimed to characterize changes in secretion of proteins from basolateral compartment of renal tubular epithelial cells after exposure to COM crystals and then examined functional significance of these changes in association with the stone pathogenesis, particularly in renal interstitium, i.e. crystal invasion and inflammatory response.

## Results

### Screening for optimal time-point of secretome study in polarized MDCK cells

**Figure 1:**
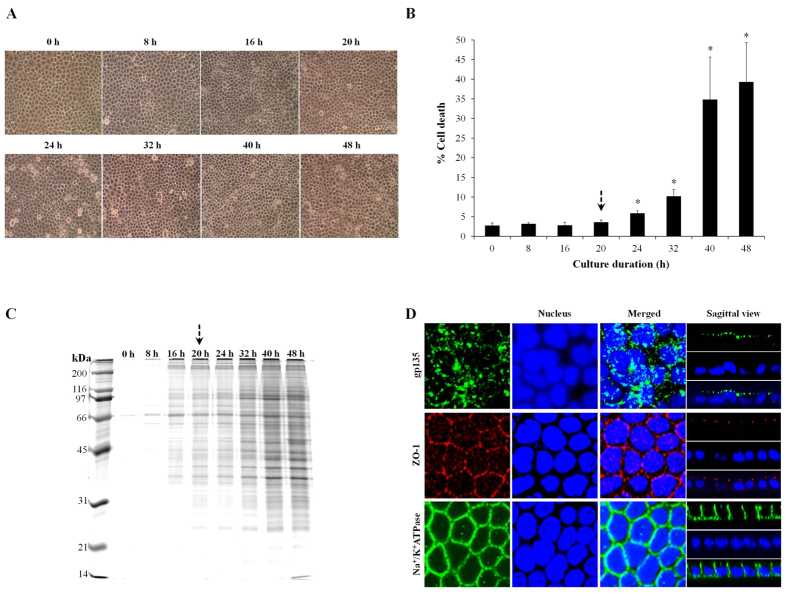
Optimal time-point for secretome analysis of polarized MDCK cells in serum-free medium. Polarized MDCK cells were maintained in serum-free medium for various time-points, i.e. 0–48 h. Morphological examination was performed under a light microscope (**A**) (original magnification was 400X), whereas cell death analysis was done by using Trypan blue staining (**B**) (n = 3 independent experiments for each bar and *represents p < 0.05 as compared to 0 h). Proteins recovered from the culture supernatant of varying time-points (with an equal volume of 1 ml) were resolved by 12% SDS-PAGE and visualized with CBB-G250 staining (**C**). An arrow indicates the optimal time-point selected for secretome analysis (the longest duration that cell death remained unchanged – to ensure that secretome was analyzable whereas severe cytotoxicity could be excluded). Finally, immunofluorescence staining of apical (gp135), tight junction (ZO-1), and basolateral (Na^+^/K^+^-ATPase) markers was performed to confirm that the cells cultivated in serum-free medium for 20 h (optimal time-point) remained polarized and intact (**D**).

To screen for the optimal time-point for subsequent secretome analysis, polarized MDCK cells were maintained in serum-free condition for up to 48 h. Morphological examination showed no obvious changes in cell morphology for up to 20 h, whereas longer incubation was associated with characteristics of cell death including rounding, increased granularity and floating (Fig. 1A,B). In addition, we also ensured that secreted proteins at individual time-points were analyzable (with sufficient amount of proteins to be analyzed subsequently). Figure 1C demonstrated that the secreted proteins were detectable from 8 h after treatment and the protein amounts were gradually increased when the incubation was prolonged. Based on morphology, cell death and amounts of secreted proteins reported in Fig. 1A–C, respectively, we selected 20 h as the optimal time-point for subsequent secretome study of polarized MDCK cells under the serum-free condition. Finally, we confirmed that MDCK cells under a starving condition at the optimal time-point remained polarized and intact (Fig. 1D).

### Effects of COM crystals on secretome from basolateral compartment of the polarized MDCK cells

**Figure 2:**
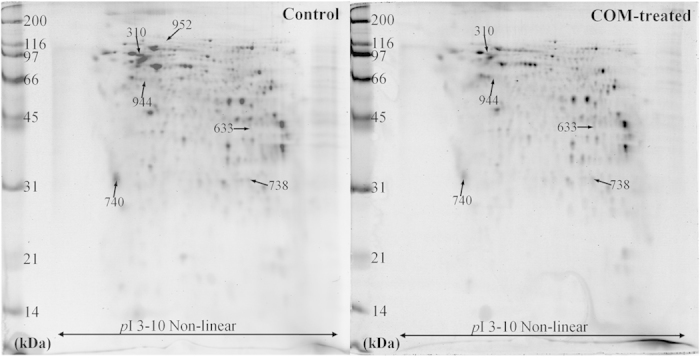
2-D map of changes in basolateral secretome induced by COM crystals. Proteins derived from lower chamber of Transwell (or basolateral compartment of polarized cells) with equal amount of 80 μg were resolved by 2-DE and visualized with Deep Purple staining. Spot matching and intensity analysis revealed significant differences in levels of six protein spots (labeled with spot numbers) between the controlled and COM-treated groups. These proteins were then identified by Q-TOF MS/MS analysis (see details in Table 1). N = 5 independent gels in each group (a total of 10 gels were analyzed).

At the optimal time-point (selected as aforementioned), secreted proteins were recovered from lower chamber of Transwell and equal amount of total proteins (80 μg) was resolved in each 2-D gel (n = 5 per group). Using Deep Purple protein dye, a total of 397 ± 42 protein spots were detected on each 2-D gel (Fig. 2). Spot matching and quantitative intensity analysis revealed significant changes in levels of six protein spots in basolateral secretome of COM-treated cells. From these, two were increased, three were decreased and one was absent in the basolateral secretome of COM-treated cells. These differentially secreted proteins were successfully identified by Q-TOF MS/MS analysis. Their identities, MS/MS identification scores, sequence coverage, number of matched peptides, and quantitative data are summarized in Table 1. These included the increased levels of two metabolic enzymes enolase-1 (spot #633) and phosphoglycerate mutase 1 (spot #738), both of which were involved mainly in glycolysis. In contrast, two cytoskeletal proteins actinin (spot #310) and alpha-tubulin 2 (spot #944), as well as a signaling protein 14-3-3 protein epsilon (spot #740) were decreased in their secretion, whereas another metabolic enzyme involved in ubiquitin-proteasome pathway, ubiquitin-activating enzyme E1 (spot #952), was absent in the basolateral secretome of the COM-treated cells (Table 1).

### Validation of the proteomic data

**Figure 3:**
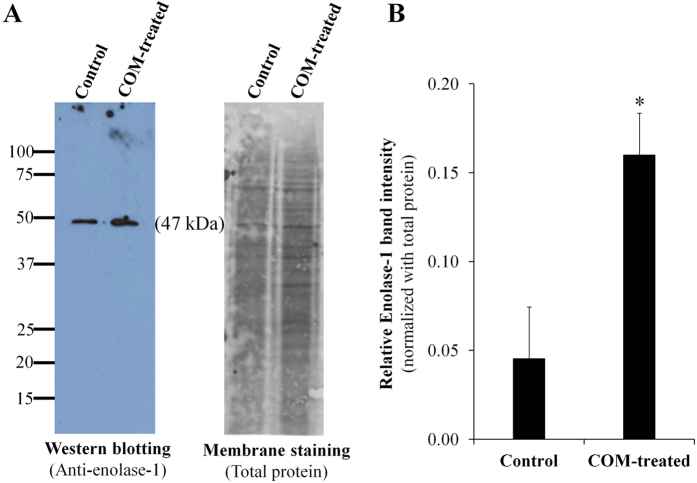
Validation of the proteomic data. The increased level of secreted enolase-1 in COM-treated group determined by proteomic analysis was confirmed by Western blot analysis. Enolase-1 band was detected using rabbit polyclonal anti-enolase-1 antibody, whereas Deep Purple was used for membrane staining to normalize band intensity. N = 3 independent experiments; *p < 0.05 vs. control.

We selected a protein that had a high-degree of changes (particularly the increase one) and had a potential role in kidney stone formation for subsequent validation. From the six significantly altered secreted proteins, alpha-enolase (enolase-1) (spot #633) had approximately 5.34-fold increased level and might have a potential role in kidney stone formation (see more details in the subsequent Discussion). We thus confirmed the increase of enolase-1 secretion in basolateral compartment of MDCK polarized cells after exposure to COM crystals. Western blot analysis confirmed that the level of secreted enolase-1 was significantly increased in the COM-treated cells (Fig. 3).

### Effect of enolase-1 on COM crystal invasion

**Figure 4:**
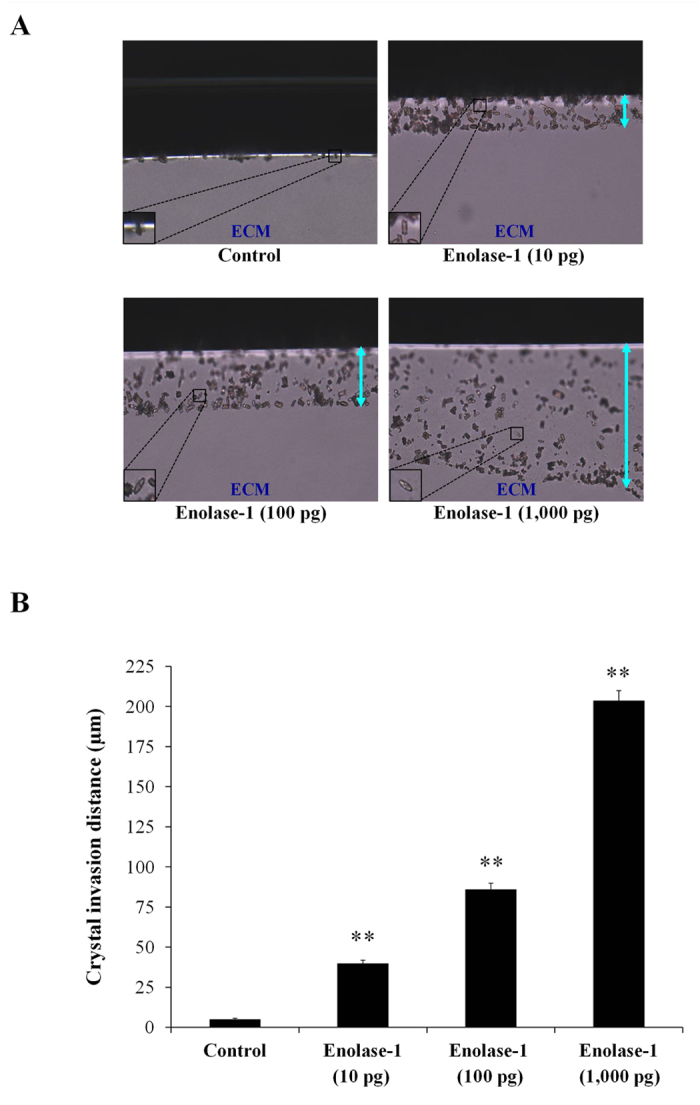
Effect of enolase-1 on COM crystal invasion. A recently established crystal invasion assay was employed to examine effects of purified enolase-1 (at various doses of 0, 10, 100 and 1,000 pg) on COM crystal invasion (**A**) (Original magnification = 100X). Crystal invasion distance (indicated by up down arrow) in the ECM migrating chamber was measured and compared (**B**). N = 3 independent experiments; **p < 0.01 vs. negative control.

Because enolase-1 was one of the COM crystal-binding proteins identified in our previous study^9^ and is known as a plasminogen-binding protein^10^, we hypothesized that it might have an important role in crystal invasion through renal interstitium. We thus employed a crystal invasion assay established by our group recently^11^ (more details can be found in Materials and Methods) to confirm such hypothesis. The result showed that enolase-1 markedly induced crystal invasion in a dose-dependent manner (from 10 to 1,000 pg) (Fig. 4).

### Binding of purified enolase-1 on U937 cell surface

**Figure 5:**
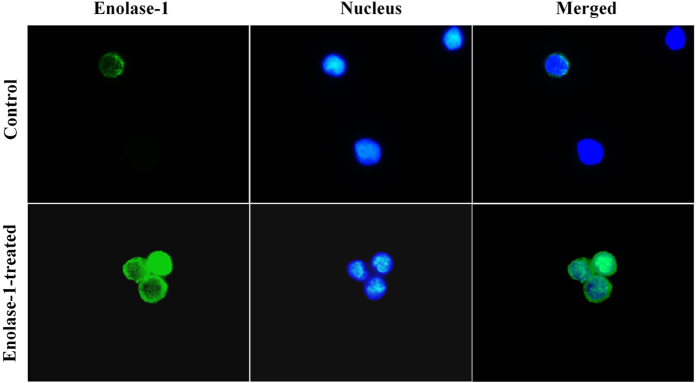
Binding of exogenous enolase-1 on U937 monocytic cell surface. U937 monocytes were incubated with 500 pM purified enolase-1 for 24 h and then washed with PBS. Surface expression of enolase-1 was then examined by immunofluorescence staining (without permeabilization) using rabbit polyclonal anti-enolase-1 as the primary antibody. Chicken anti-rabbit IgG antibody conjugated with AlexaFluor-488 (green) served as the secondary antibody, whereas Hoechst dye (blue) was used for nuclear staining. The images were captured under a fluorescent microscope (Original magnification power = 1,000X).

We further hypothesized that, in addition to crystal invasion, the increased enolase-1 secretion in basolateral compartment might have a crucial role in migration of inflammatory cells, i.e. monocytes, into renal interstitium to aggravate inflammatory cascade. We thus confirmed that secreted enolase-1 was able to bind to the cellular surface of monocytes. The binding assay and immunofluorescence imaging revealed that purified enolase-1 could bind to the surface of U937 monocytes (Fig. 5).

### Effect of enolase-1 on monocytes migration

**Figure 6:**
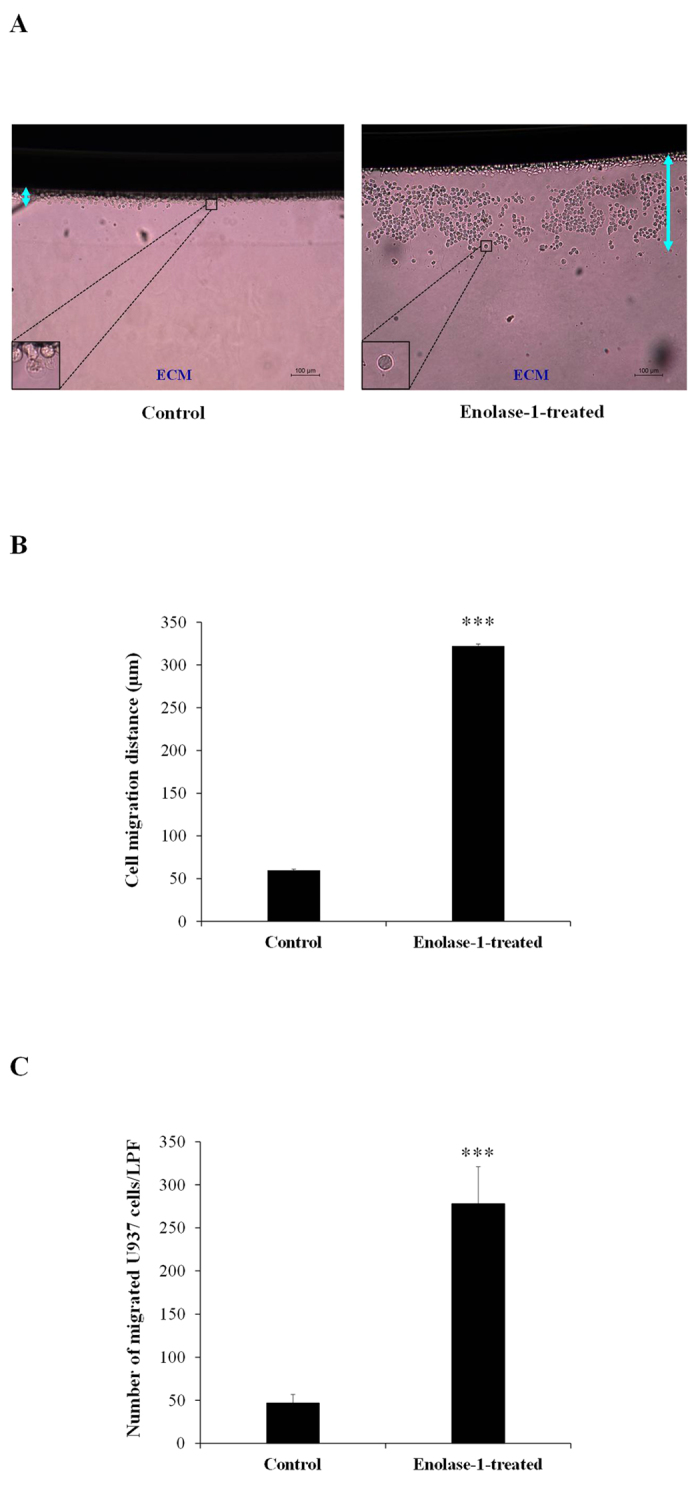
Effect of increased surface enolase-1 on U937 monocytic cell migration through ECM migrating chamber. After U937 cells were bound with purified enolase-1, the cells with or without enolase-1 bound were subjected to cell migration assay using ECM migration chamber (as detailed in “Materials and Methods”) (**A**) (Original magnification = 100X). Cell migration distance (indicated by up down arrow in panel (**A**) (**B**) and number of the migrated cells (**C**) were then quantitated and compared. N = 3 independent experiments; ***p < 0.001 vs. control.

Finally, we confirmed that the bound enolase-1 on monocytes’ cell surface could induce monocytic cell migration through renal interstitium using the ECM migrating chamber. Methods used for cell migration assay through ECM migrating chamber were similar to those used for COM crystal invasion assay with some modifications by replacing the crystals with U937 monocytic cells (details are provided in Materials and Methods). The data showed marked increase in both cell migration distance and number of the migrated monocytic cells through the ECM migrating chamber for the cells coated with purified enolase-1 when compared to the uncoated U937 cells, which served as the control (Fig. 6).

## Discussion

Apical and basolateral compartments of polarized epithelial cells require protein sorting and trafficking mechanisms for compartment-specific protein transport and secretion to maintain homeostasis^12^,^13^. Changes in this physiologic control, thus, can be associated with some diseases^1^. In this study, we have proposed that changes in secretion of proteins from basolateral compartment of renal tubular epithelial cells induced by COM crystals are associated with kidney stone pathogenic mechanisms, specifically crystal invasion into renal interstitium and subsequent inflammatory process.

MDCK (Type II) cell line was used as a cell model in this study because of its properties, including i) stable epithelial phenotype, ii) lack of gap junctions, and iii) large and tall cell morphology, making this cell line suitable for secretome study^14^. These properties lead to a clear separation of apical and basolateral compartments and are comparable to the phenotypes of epithelial cells lining along renal tubules *in vivo*. Moreover, this cell line has been widely employed in several previous studies on apical and basolateral secretomes^15^,^16^,^17^.

COM crystal at a concentration of 100 μg (crystal)/ml (medium) was selected in this study because this dosage is sufficient to trigger cellular response without any significant changes of cell death (both apoptosis and necrosis) as demonstrated in our previous study^7^. In order to evaluate changes in secretome or secreted proteins *in vitro*, one factor that needs to be avoided is contamination of proteins from serum that is used for routine cell cultivation^1^,^18^,^19^. We thus carefully screened for an optimal time-point to ensure that the polarized MDCK cells could be maintained in a serum-free condition for a certain period that cells remained polarized and intact (as we intended to investigate response of the cells to COM crystals but did not want to examine changes caused by severe cytotoxicity or cell death). To confirm the polarization status of the treated cells, we evaluated expression of three representative markers for apical membrane (gp135)^20^, tight junction (ZO-1)^21^, and basolateral membrane (Na^+^/K^+^-ATPase)^22^. The data revealed that such optimal time-point was 20-h after exposure to COM crystals, at which the cells remained polarized and healthy with considerable amount of secreted proteins for subsequent analysis (Fig. 1).

Secretome analysis using 2-D gel-based proteomics approach revealed significant changes in levels of six proteins secreted into basolateral compartment (or lower chamber of the Transwell) (Fig. 2 and Table 1). It was not unexpected that only a small number of secreted proteins were detected with significant changes in their levels after COM crystal intervention by the following reasons. First, we carefully selected an optimal condition (i.e. COM crystal dosage and exposure time-point in serum-free environment) as discussed above to make sure that changes to be observed were not from cytotoxic effects but indeed were from cellular response to the crystal stimulus. Second, high-stringency criteria were used for spot detection (high-saliency parameter setting) and statistical analysis were very helpful to screen out the potentially false positive data and those with high-degree variations among gels within the same group. Finally, we used a gel-based proteomics approach of which sensitivity is relatively low as compared to the gel-free techniques. Using more advanced and higher sensitive technologies would yield a larger number of significantly altered secreted proteins induced by COM crystals.

From these differentially secreted proteins, we focused our attention to the increase of enolase-1, which is also known as a COM crystal-binding protein^9^ and a plasminogen-binding protein^10^. Indeed, our previous study identified a decreased level of whole cell enolase-1 under a response to treatment with 1,000 μg/ml COM crystals for 48-h^8^. In should be noted that this discrepancy (increase of secreted enolase-1 in the present study vs. decrease of whole cell enolase-1 in the previous study) was not surprising as there were some differences in the study design in these two studies: First, the dosage of COM crystals used (non-cytotoxic vs. cytotoxic dose in the present vs. previous study, respectively); Second, the crystal exposure time (20 vs. 48 h in the present vs. previous study, respectively); and Finally, the cellular compartment to be examined (secretion vs. whole cell in the present vs. previous study, respectively). These data implicated that effects of COM crystals were dose- and time-dependent, and compartment-specific.

Enolase-1 is a multifunctional protein that plays roles in several cellular functions^23^. Enolase-1 also serves as a plasminogen receptor on the cell surface of hematopoetic, epithelial and endothelial cells involving in fibrinolytic process^10^,^23^. Its octameric structure can bind to plasminogen at the C-terminal lysine (433^th^ residue) and/or putative residues (248^th^–256^th^)^24^. Previous studies have shown that enolase-1 on microbial cell surfaces can promote invasion and migration properties of the microbes in host or target cells via binding to plasminogen^10^,^25^. Because crystal invasion is a crucial step of kidney stone formation and enolase-1 can bind to both COM crystal^9^ and plasminogen^10^ and then activate plasmin activity to degrade ECM, we have thus hypothesized that enolase-1 is one of the important secreted proteins that triggers crystal invasion through the renal interstitium using its capability to bind to both COM crystal and plasminogen. Crystal invasion assay confirmed that enolase-1 had a dramatic effect to promote COM crystal invasion through the ECM migrating chamber in a dose-dependent manner (Fig. 4). Note that the crystal invasion assay is quite specific as we have demonstrated that only some limited number of proteins, not all, had a positive result^11^.

To further elucidate the important role of enolase-1 in kidney stone pathogenesis, we addressed its role in association with monocytes that could subsequently trigger inflammatory response in the renal interstitium^26^. In a previous study, enolase-1 has been demonstrated to promote plasminogen-mediated recruitment of monocytes to the inflamed lungs^27^. In bacteria, enolase-1 has been demonstrated to be a protein bound onto the bacterial cell surface that can subsequently activate tissue invasion^25^,^28^. However, there was no evidence demonstrating that secreted enolase-1 can bind to mammalian cells, particularly inflammation-associated cells such as monocytes. We thus confirmed that purified (representing secretory form of) enolase-1 could bind to the cell surface of U937 monocytes (Fig. 5). Moreover, the monocytes bound with enolase-1 had dramatic migratory activity through ECM migrating chamber as compared to the controlled cells without enolase-1 binding (Fig. 6).

In summary, we have successfully identified six proteins whose basolateral secretion levels were significantly altered when the polarized MDCK cells were exposed to COM crystals for 20 h. From these, the increased level of enolase-1 was confirmed by Western blot analysis and its significant role in kidney stone formation was addressed. Functional validation showed that enolase-1 could dramatically induce COM crystal invasion through ECM migrating chamber and could bind to the cell surface of human monocytes. Finally, enolase-1 bound onto the monocytic cell surface could activate monocytic cell migration through ECM migrating chamber. Taken together, our data indicate that when renal tubular epithelial cells are exposed to COM crystals, the cells secrete greater level of enolase-1 into basolateral compartment, which in turn promotes COM crystal invasion and monocytic cell migration through renal interstitium that can subsequently aggravate inflammatory response as well as kidney stone formation. These data provide an important basis for further elucidation of sophisticated pathogenic mechanisms of kidney stone disease.

## Materials and Methods

### Cell cultivation and polarization

High-passage parental Mardin-Darby Canine kidney (MDCK) cell line (ATCC) (which displayed many characteristics of MDCK Type II) (approximately 2 × 10^6^ cells) was seeded and grown on pre-wetted collagen type IV (Sigma; St. Louis, MO) coated polycarbonate membrane Transwell^TM^ insert (0.4 μm pore size, Corstar; Cambridge, MA) in Eagle’s minimum essential medium (MEM) supplemented with 10% fetal bovine serum (FBS) (Gibco, Invitrogen Corporation; Grand Island, NY), 2 mM L-glutamine and 1.2% penicillin G/streptomycin (Gibco) for 2 days in a humidified incubator with 5% CO_2_ at 37 °C. Thereafter, the cultured medium was discarded and the cells were washed gently with PBS 5 times to remove serum. The cells were then maintained in serum-free MEM medium and further incubated in a humidified incubator with 5% CO_2_ at 37 °C for up to 48 h. Microscopic examination was performed at 0, 8, 16, 20, 24, 32, 40 and 48 h after cultivation of the cells in serum-free medium using a CKX41 microscope (Olympus Co. Ltd.; Tokyo, Japan).

### Trypan blue cell death assay

Cell death was quantitated during 0–48 h cultivation in serum-free medium. Briefly, all the cells (both adhered and floated cells) were collected by trypsinization using 0.1% trypsin in 2.5 mM EDTA. The collected cells in each time-point were stained with trypan blue and percentage of cell death was determined.

### SDS-PAGE (1-DE) of secreted proteins

Serum-free culture supernatants were collected from lower chamber of Transwell^TM^ (basolateral compartment) after cultivation in serum-free medium for 0–48 h (with an equal volume of 1 ml). The collected supernatants were then dialyzed against deionized water and lyophilized. The recovered proteins were resuspended in Laemmli’s buffer, resolved by 12% SDS-PAGE and then visualized with Coomassie Brilliant Blue (CBB)-G250 (USB; Cleveland, OH) to observe protein band pattern and amount of secreted proteins.

### Immunofluorescence staining of apical, tight junction, and basolateral markers

To confirm that the cells cultivated in serum-free medium for 20 h, which was selected as the optimal time-point for subsequent secretome analysis in the present study, remained polarized and intact, the polarized MDCK monolayer was washed with membrane preserving buffer (1 mM MgCl_2_ and 0.1 mM CaCl_2_ in PBS), and then fixed with 3.7% formaldehyde in PBS at 25 °C for 10 min and permeabilized with 0.1% Triton X-100 at 25 °C for another 10 min. After extensive washing with membrane preserving buffer, MDCK cells were incubated with rabbit polyclonal anti-gp135 (apical membrane marker) (Santa Cruz Biotechnology; Santa Cruz, CA), mouse monoclonal anti-zonula occludens-1 (ZO-1) (tight junction marker) (Invitrogen-Molecular Probes; Burlington, ON, Canada), or mouse monoclonal anti-Na^+^/K^+^-ATPase-α1 subunit (basolateral membrane marker) (Santa Cruz Biotechnology) antibody (with a dilution of 1:50 in 1% BSA/PBS for all) at 37 °C for 1 h. The cells were then rinsed with PBS three times and then incubated with respective secondary antibody conjugated with rhodamine or AlexaFluor-488 (Invitrogen-Molecular Probes) (1:5,000 in 1% BSA/PBS) containing 0.1 μg/ml Hoechst dye (DNA staining for nuclear localization) (Invitrogen-Molecular Probes) at 37 °C for 1 h. Thereafter, the cells were examined by a laser-scanning confocal microscope equipped with an LSM5 Image Browser (LSM 510 Meta, Carl Zeiss; Jena, Germany).

### Treatment with COM crystals and protein preparation

COM crystals were prepared and sterilized as previously described^29^. This preparation method consistently provided typical monoclinic prismatic shape of the crystals with a size of 5–10 μm as has been demonstrated by both light and scanning electron microscopes in our previous study^7^. The polarized MDCK cells were maintained in serum-free medium with or without 100 μg/ml COM crystals for 20 h (n = 5 independent cultures per group). Thereafter, serum-free culture supernatants were collected from lower chamber of Transwell^TM^. The collected supernatants were then dialyzed against deionized water and lyophilized. The recovered proteins were resuspended in a 2-D lysis buffer containing 7 M urea, 2 M thiourea, 4% 3-[(3-cholamidopropyl)dimethylammonio]-1-propanesulfonate (CHAPS), 2% (v/v) ampholytes (pH 3–10), 120 mM dithiothreitol (DTT) and 40 mM Tris-base. Protein concentrations were quantitated by Bradford’s method using Bio-Rad Protein Assay (Bio-Rad Laboratories; Hercules, CA).

### Two-dimensional gel electrophoresis (2-DE)

Equal amount (80 μg total protein) of secreted proteins from basolateral compartment was rehydrated on each Immobiline DryStrip (non linear pH gradient of 3–10, 7-cm long; GE Healthcare, Uppsala, Sweden) overnight (n = 5 individual strips per group). The secreted proteins were then separated in the first dimension by isoelectric focusing (IEF) using Ettan IPGphor III system (GE Healthcare) at 20 °C with a stepwise mode to reach 9,083 Vh with a limited current of 50 μA/strip. After IEF, the strips were equilibrated in equilibration buffer I containing 6 M urea, 130 mM DTT, 112 mM Tris-base, 4% SDS, 30% glycerol and 0.002% bromphenol blue for 15 min and then in equilibration buffer II containing 6 M urea, 135 mM iodoacetamide, 112 mM Tris-base, 4% SDS, 30% glycerol and 0.002% bromphenol blue. Second dimensional separation was then performed by 13% SDS-PAGE at 150 V for approximately 2 h. The resolved protein spots were then visualized by Deep Purple protein staining (GE Healthcare) and then imaged by Typhoon laser scanner (GE Healthcare) with an excitation of 532 nm and emission of 610-nm band-pass filter. The photo-multiplier tube (PMT) was tuned to 600 V to obtain the resolution at 100-μm pixel size.

### Spot matching and quantitative intensity analysis

Protein spots visualized in 2-DE gels were analyzed using ImageMaster 2D Platinum software (GE Healthcare). Parameters used for spot detection were (i) minimal area = 10 pixels; (ii) smooth factor = 2.0; and (iii) saliency = 500. A reference gel selected from all 2-DE gels was used for determination of existence and difference of protein expression between gels according to the manufacturer’s instructions. Intensity volumes of individual spots relative to total intensity of all spots in each gel were obtained and subjected to statistical analysis. Differentially expressed protein spots that reached statistically significant threshold (p < 0.05) were subjected to in-gel tryptic digestion and identification by mass spectrometry.

### In-gel tryptic digestion

The protein spots with significantly differential levels were excised from 2-D gels, washed twice with 200 μl of 50% acetonitrile (ACN)/25 mM NH_4_HCO_3_ buffer (pH 8.0) at RT for 15 min, and then washed once with 200 μl of 100% ACN. After washing, the solvent was removed, and the gel pieces were dried by a SpeedVac concentrator (Savant; Holbrook, NY) and rehydrated with 10 μl of 1% (w/v) trypsin (Promega; Madison, WI) in 25 mM NH_4_HCO_3_. After rehydration, the gel pieces were crushed and incubated at 37 °C for at least 16 h. Peptides were subsequently extracted twice with 50 μl of 50% ACN/5% trifluoroacetic acid (TFA) and the extracted solutions were then combined and dried with the SpeedVac concentrator. The peptide pellets were resuspended with 10 μl of 0.1% TFA and purified using ZipTip_C18_ (Millipore; Bedford, MA). The peptide solutions were drawn up and down in the ZipTipC_18_ ten times and then washed with 10 μl of 0.1% formic acid by drawing up and expelling the washing solution three times. The peptides were finally eluted with 5 μl of 75% ACN/0.1% formic acid.

### Protein identification by quadrupole time-of-flight tandem mass spectrometry (Q-TOF MS/MS)

The trypsinized samples were premixed 1:1 with the matrix solution containing 5 mg/ml α-cyano-4-hydroxycinnamic acid (CHCA) in 50% ACN, 0.1% (v/v) TFA and 2% (w/v) ammonium citrate, and deposited onto the 96-well MALDI target plate. The samples were analyzed by Q-TOF Ultima^TM^ mass spectrometer (Micromass; Manchester, UK), which was fully automated with predefined probe motion pattern and the peak intensity threshold. Within each sample well, parent ions that met the predefined criteria (any peak within the *m/z* 800–3,000 range with intensity above 10 count ± include/exclude list) were selected for CID MS/MS using argon as the collision gas and a mass dependent ±5 V rolling collision energy until the end of the probe pattern was reached. The MS/MS data were extracted and outputted as the searchable *.pkl* files, for independent searches using the MASCOT search engine (http://www.matrixscience.com) to query against the NCBI mammalian protein database, assuming that peptides were monoisotopic. Fixed modification was carbamidomethylation at cysteine residues, whereas variable modification was oxidation at methionine residues. Only one missed trypsin cleavage was allowed, and peptide mass tolerances of 50 ppm were allowed for MS/MS ions search.

### Western blot analysis

Western blot analysis was performed to confirm the proteomic data. Secreted proteins from basolateral compartment of controlled or COM-treated polarized MDCK cells were resolved in 12% SDS-PAGE gel with an equal amount of total proteins (30 μg) in each lane. The separated proteins were transferred onto a nitrocellulose membrane where non-specific bindings were subsequently blocked with 5% skim milk in PBS at RT for 1 h. The membrane was incubated with rabbit polyclonal anti-enolase-1 antibody (Santa Cruz Biotechnology) (1:1,000 in 1% skim milk/PBS) at 4 °C overnight. After washing with PBS three times, the membrane was incubated with swine anti-rabbit IgG conjugated with horseradish peroxidase (1:2,000 in 1% skim milk/PBS; DAKO Glostrup, Denmark) at RT for 1 h. Immunoreactive bands were developed by SuperSignal West Pico chemiluminescence substrate (Pierce Biotechnology; Rockford, IL) and were then visualized by autoradiogram.

### Crystal invasion assay

Crystal invasion assay was performed according to our recently established protocol^11^. Briefly, a total of 20 μg COM crystals was added into 200 μl of MEM. Then, various amounts (0, 10, 100, and 1,000 pg) of purified enolase-1 (Sino Biological Inc.; Beijing, China) was added and the mixture was incubated at 4 °C overnight with gently continuous agitation. The unbound protein was discarded by a centrifugation at 15,000 rpm and 4 °C for 5 min, and the crystal-protein complex was washed with PBS. Thereafter, 200 μl of 0.3 pM Lys-plasminogen (Fitzgerald Industries international; Acton, MA) in PBS was mixed and incubated with the crystal-protein complex at 37 °C for 1 h. The unbound plasminogen was discarded by a centrifugation at 15,000 rpm for 5 min and the pellet was washed with PBS once. Then, 100 μl of 0.15 pM urokinase plasminogen activator (uPA) (Fitzgerald Industries International) in PBS was mixed with the crystal-protein-plasminogen complex. The mixture was then added on-top of the matrix gel inside the extracellular matrix (ECM) migration chamber and incubated at 37 °C for 24 h. After 24-h incubation, the solution remained on the upper part of the migration chamber was removed by absorption using a gauze or tissue paper. The invaded COM crystals inside the matrix gel were then imaged using a light microscope with differential interference contrast (DIC) mode (Nikon H600L, Nikon Corp.; Tokyo, Japan). The crystal invasion distance was measured and averaged from at least 15 different fields within the same chamber using Image Frame Work software version 0.9.6 (Tarosoft^®^; Nonthaburi, Thailand). For the control, COM crystals were processed as aforementioned without binding protein, but with the presence of plasminogen and uPA at later steps.

### Binding of purified enolase-1 on U937 cell surface and immunofluorescence staining

U937 monocytes were maintained in RPMI 1640 supplemented with 10% FBS, 1.2% (v/v) penicillin G/streptomycin (Gibco, Invitrogen Corporation) in a humidified incubator with 5% CO_2_ at 37 °C. The cells were then incubated with or without 500 pM purified enolase-1 (Sino Biological Inc.) for 24 h. Thereafter, the cells were collected by a centrifugation at 1,500 rpm and 4 °C for 5 min. The cell pellets were washed with PBS once and fixed with 3.7% formaldehyde/PBS at room temperature for 10 min (without permeabilization). The fixed cells were then probed with rabbit polyclonal anti-enolase-1 antibody (Santa Cruz Biotechnology) at a dilution of 1:50 in 1% BSA/PBS at 37 °C for 1 h. Thereafter, the cells were incubated with chicken anti-rabbit IgG conjugated with Alexa 488 (Invitrogen-Molecular Probes; Burlington, ON, Canada) at a dilution of 1:2,000 in 1% BSA/PBS for 1 h. Nuclei were counterstained with 0.1 μg/ml Hoechst dye (Invitrogen; Paisley, UK). The coverslip was then mounted onto a slide using 50% glycerol/PBS and the fluorescence images were captured under ECLIPSE 80i fluorescence microscope (Nikon). Immunofluorescence intensity levels were measured using ImageJ software (version 1.48b) (http://imagej.nih.gov/ij/).

### Cell migration assay through ECM migrating chamber

Approximately 5 × 10^4^ U937 cells (with or without enolase-1 binding as detailed above) were incubated with 15 nM Lys-plasminogen (Fitzgerald Industries international) in RPMI 1640 medium (Gibco) at 37 °C in a humidified incubator with 5% CO_2_ for 1 h. The unbound plasminogen was discarded by a centrifugation at 1,500 rpm for 5 min and the cells were resuspended in 100 μl RPMI 1640 medium (Gibco). Thereafter, 3 nM urokinase plasminogen activator (uPA) (Fitzgerald Industries International) was mixed with the cell-enolase-1-plasminogen complex. The mixture was then overlaid on-top of the matrix gel inside the ECM migration chamber and incubated at 37 °C in a humidified incubator with 5% CO_2_ for 24 h. After 24-h incubation, the cells remained on the upper part of the migration chamber were removed by absorption using a gauze or tissue paper. The migrated cells inside the matrix gel were then imaged using a light microscope with a DIC mode (Nikon H600L, Nikon Corp.). The cell migration distance was measured and averaged from at least 15 different fields within the same chamber using Image Frame Work software version 0.9.6 (Tarosoft^®^), whereas the number of the migrated cells was counted .

### Statistical analysis

Unless stated otherwise, all quantitative/comparative experiments were performed in triplicates. All quantitative data are reported as mean ± SEM. Comparisons of the quantitative data were performed by unpaired Student’s *t*-test using SPSS software ,version 13.0 (SPSS; Chicago, IL, USA). *P* values less than 0.05 were considered statistically significant.

## Additional Information

**How to cite this article**: Chiangjong, W. and Thongboonkerd, V. Calcium oxalate crystals increased enolase-1 secretion from renal tubular cells that subsequently enhanced crystal and monocyte invasion through renal interstitium. *Sci. Rep.* **6**, 24064; doi: 10.1038/srep24064 (2016).

## Figures and Tables

**Table 1:** Summary of secreted proteins whose levels were significantly altered after the polarized MDCK cells were exposed to COM crystals.

Spot no.	Protein name	NCBI ID	MS/MS identification scores	% Cov	No. of matched peptides	p*I*	MW (kDa)	Intensity (Mean ± SEM)		Ratio (COM-treated/Control)	*p*values
								Control	COM-treated		
Secreted proteins whose levels were significantly increased in basolateral part of COM treated polarized MDCK cells											
633	Alpha enolase (Enolase 1) (2-phospho-D-glycerate hydrolyase) (Non-neural enolase) (Phosphopyruvate hydratase) (C-myc promoter-binding protein) (MBP-1) (MPB-1) (Plasminogen-binding protein) isoform 1	gi|73956716	895	41	23	6.57	45.47	0.0439 ± 0.0276	0.2342 ± 0.0467	5.34	0.0080
738	Phosphoglycerate mutase 1 (Phosphoglycerate mutase isozyme B) (PGAM-B) (BPG-dependent PGAM 1) isoform 1	gi|57107225	415	30	8	6.67	28.92	0.1748 ± 0.0489	0.4379 ± 0.0706	2.50	0.0156
Secreted proteins whose levels were significantly decreased in basolateral part of COM treated polarized MDCK cells											
310	Actinin, alpha 4 isoform 2	gi|73947718	1813	43	39	5.27	105.32	0.5986 ± 0.1094	0.2435 ± 0.0774	0.41	0.0293
740	14-3-3 Protein epsilon (14-3-3E) (Mitochondrial import stimulation factor L subunit) (MSF L) isoform 1	gi|73967154	600	48	15	4.77	27.57	3.1557 ± 0.2683	1.9139 ± 0.2747	0.61	0.0120
944	Tubulin alpha-2 chain (Alpha-tubulin 2) isoform 2	gi|73996516	380	24	9	4.97	50.56	0.2526 ± 0.0575	0.0314 ± 0.0314	0.12	0.0097
Secreted protein whose levels was absent in basolateral part of COM treated polarized MDCK cells											
952	Ubiquitin-activating enzyme E1	gi|74007348	876	21	18	5.87	113.46	0.0233 ± 0.0098	0.0000 ± 0.0000	0.00	0.0444

NCBI = National Center for Biotechnology Information.

% Cov = % Sequence coverage [(number of the matched residues/total number of residues in the entire sequence) × 100%].
